# A mathematical model of plasmin-mediated fibrinolysis of single fibrin fibers

**DOI:** 10.1371/journal.pcbi.1012684

**Published:** 2024-12-20

**Authors:** Roukayatou R. Ouedraogo, Hannah K. Sowers, Spencer R. Lynch, Nathan E. Hudson, Brittany E. Bannish

**Affiliations:** 1 Department of Mathematics and Statistics, University of Central Oklahoma, Edmond, Oklahoma, United States of America; 2 Department of Physics, East Carolina University, Greenville, North Carolina, United States of America; University of Michigan, UNITED STATES OF AMERICA

## Abstract

Fibrinolysis, the plasmin-mediated degradation of the fibrin mesh that stabilizes blood clots, is an important physiological process, and understanding mechanisms underlying lysis is critical for improved stroke treatment. Experimentalists are now able to study lysis on the scale of single fibrin fibers, but mathematical models of lysis continue to focus mostly on fibrin network degradation. Experiments have shown that while some degradation occurs along the length of a fiber, ultimately the fiber is cleaved at a single location. We built a 2-dimensional stochastic model of a fibrin fiber cross-section that uses the Gillespie algorithm to study single fiber lysis initiated by plasmin. We simulated the model over a range of parameter values to learn about patterns and rates of single fiber lysis in various physiological conditions. We also used epifluorescent microscopy to measure the cleavage times of fibrin fibers with different apparent diameters. By comparing our model results to the laboratory experiments, we were able to: 1) suggest value ranges for unknown rate constants(namely that the degradation rate of fibrin by plasmin should be ≤ 10 s^−1^ and that if plasmin crawls, the rate of crawling should be between 10 s^−1^ and 60 s^−1^); 2) estimate the fraction of fibrin within a fiber cross-section that must be degraded for the fiber to cleave in two; and 3) propose that that fraction is higher in thinner fibers and lower in thicker fibers. Collectively, this information provides more details about how fibrin fibers degrade, which can be leveraged in the future for a better understanding of why fibrinolysis is impaired in certain disease states, and could inform intervention strategies.

## Author summary

Fibrinolysis, the enzymatic degradation of the fibrin fibers that stabilize blood clots, plays an important role in preventing heart attacks and strokes during wound healing. Because blood clots exhibit multiscale structural features, a wholistic understanding of clot degradation requires knowledge of how single enzymatic reactions propagate through the larger-scale clot features. Limitations of experimental techniques typically restrict their results to one spatial scale, so mathematical models have the potential to explain the origins of experimental results and act as a bridge that connect results across spatial scales. Recent experimental results have probed mechanisms regulating the digestion of individual fibrin fibers, however mathematical models of fibrinolysis continue to focus mostly on the degradation of full fibrin clots. We built a 2-dimensional stochastic fibrinolysis model to interrogate the digestion of single fibrin fibers by plasmin, the primary fibrinolytic enzyme. By comparing our model results to laboratory experiments of fiber degradation, we estimated previously unmeasurable lysis rates, demonstrated that plasmin likely crawls between protofibrils, and approximated the fraction of a fiber that must be degraded prior to cleavage. This foundational mathematical model could be leveraged in the future to determine mechanistic origins of certain disease states and could inform intervention strategies.

## Introduction

Blood clots are critically important in normal physiology, where they prevent excessive bleeding after blood vessel injury. Platelets, red blood cells, and other hemostatic factors are stabilized by a mesh of fibrin fibers to form the clot, which helps control bleeding. However, if blood clots are not cleared appropriately, then dangerous complications such as ischemic stroke and myocardial infarction can occur. Understanding physiological mechanisms of fibrinolysis, the enzymatic degradation of fibrin fibers by plasmin, is important for developing therapies for pathophysiological conditions.

Mathematical models have helped elucidate the basic biology of fibrinolysis and evaluate and propose new therapies [1], but most existing models of lysis focus on larger-scale degradation of the entire fibrin clot and are differential equations-based [2–9]. These models have provided important insights into macroscale fibrinolysis but cannot teach us about how lysis is happening at the single fiber scale. The development of stochastic multiscale models of fibrinolysis allowed for some information about microscale degradation [10, 11], but most results in those studies continued to focus on macroscale lysis. The model presented here is an important addition to the field as it directly studies single fiber lysis and accounts for stochastic effects that occur on the microscale. This model makes it possible, for the first time, to generate and test hypotheses about mechanisms of single fibrin fiber degradation.

Fibrin fibers are formed during clotting when thrombin cleaves fibrinogen molecules to create fibrin molecules. Each molecule contains pairs of 3 different polypeptide chains—*α*, *β*, and *γ*—which interact with other fibrin molecules in a half-staggered manner to polymerize into double-stranded protofibrils. Protofibrils laterally aggregate into fibrin fibers which can range in diameter from 20–400 nm [12]. Fibers form junctions/branches with other fibers resulting in a mesh of fibrin fibers that stabilize the blood clot. Factor XIIIa (FXIIIa) crosslinks fibrin fibers, providing even more mechanical stability [13].

Fibrin fibers are lysed by plasmin, which enzymatically degrades fibrin at discrete locations along the fiber, ultimately resulting in cleavage of the fiber at a single point [14]. It has been hypothesized, though not confirmed, that plasmin can “crawl” across a fiber, which might help explain why fibers are cut transversely [11, 15]. It has also recently been shown that cleavage is aided by a tension-dependent mechanism that pulls the fiber apart during degradation [14]. *α*2-antiplasmin (*α*2-AP) is a very strong inhibitor of free plasmin, so to be effective, plasmin must be created locally on the fibrin fibers where it is protected from *α*2-AP. This is achieved by tissue-type plasminogen activator (tPA) binding to fibrin in close proximity to bound plasminogen, at which point the tPA converts the plasminogen to plasmin. *α*2-AP is such a strong inhibitor of plasmin that the only Food and Drug Administration approved thrombolytic treatment for ischemic stroke is recombinant-tPA (not plasmin). However, since recombinant-tPA is only effective if administered within 4.5 hours of stroke onset and bleeding complications often occur [16], plasmin has still occasionally been studied for its possible use in thrombolysis [17, 18]. Plasmin is also used to initiate lysis in *in vitro* purified experiments of fibrinolysis [14, 19–22].

This paper introduces a new mathematical model of single fiber lysis initiated by plasmin. By using our stochastic model in conjunction with laboratory experiments, we gain a deeper understanding of the mechanisms underlying the fibrinolytic process. In particular, we show how single fiber cleavage times and patterns of degradation are affected by various rate constants and by the fiber diameter, and we hypothesize what fraction of fibrin within a fiber cross-section must be degraded before the fiber is cleaved.

## Materials and methods

### Stochastic two-dimensional model of plasmin-mediated fibrinolysis

We developed a stochastic 2-dimensional (2D) model of a fibrin fiber cross-section to study single fiber lysis initiated by plasmin. The model is a modified version of the Bannish et al. microscale model [10, 11], which investigated lysis initiated by tPA. To mimic laboratory experiments [14, 22] (and “Laboratory experiments” section below), the model consists of an uncrosslinked, pre-formed fibrin fiber which is exposed to plasmin. Lynch, et al. found that, on average, a 20-*μ*m-long fiber has 14 digestion sites—locations along the fiber at which degradation is occurring [14]. Hence, we choose to use a stochastic model that tracks individual plasmin molecules rather than a deterministic model which would consider plasmin concentrations.

#### Model domain

**Fig 1:**
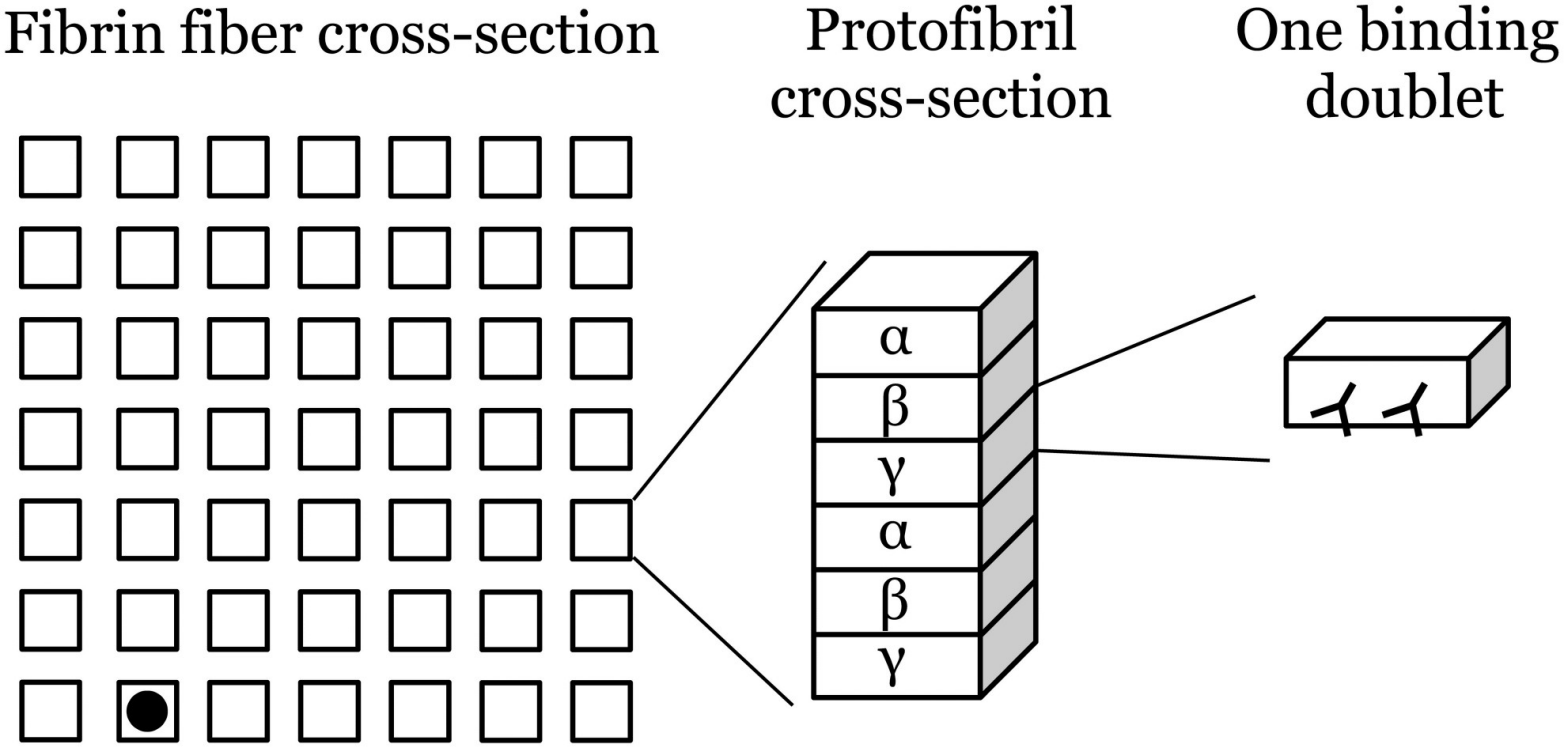
Microscale model of a fibrin fiber cross-section. The 72.7 nm diameter fibrin fiber cross-section is composed of 49 squares representing the 49 protofibril cross-sections. Each protofibril cross-section is a stack of 6 binding doublets, representing the 6 chains of a protofibril. A binding doublet is a pair of binding sites (represented by the sideways ‘Y’ shapes), to which plasmin may bind. Lysis is initiated in the model by randomly placing a plasmin molecule (black disk) on a binding doublet at a protofibril on the outer edge of the fiber.

Since fibers are cleaved at a single point along their length [14], we take the model domain to be a fiber cross-section. Degradation of fibrin within this cross-section will result in cleavage of the fiber. To simplify the numerical simulations, we assume the cross-section is a square of equal area to the circular fiber cross-section. Protofibril cross-sections are distributed uniformly throughout the fiber cross-section (Fig 1). To determine how many protofibrils, *n*, a fiber of a given diameter, *d*, should have we assume that a fibrin fiber is about 20% protein [23, 24], that a protofibril is a cylinder of solid protein, and that a protofibril has diameter 4.8 nm. The necessary number of protofibrils is then the percentage of a fiber cross-section that is protein, *p*, times the ratio of the area of the fiber cross-section to the area of the protofibril cross-section:

$$\begin{array}{c} n=\frac{p\pi {\left(\frac{d}{2}\right)}^{2}}{\pi {\left(\frac{4.8}{2}\right)}^{2}}=\frac{pd^{2}}{23.04}\approx \frac{0.2d^{2}}{23.04}\approx 0.00868d^{2}. \end{array}$$ (1)

So, for a fiber with diameter 72.7 nm, we need approximately *n* = 46 protofibrils. Since we arrange protofibrils in a square pattern throughout the square cross-section, we round this number to 49 and our domain becomes a 7 × 7 grid of protofibrils (Fig 1). Taking *n* = 49 and *d* = 72.2 in the equation above, we find that *p* = 0.21. For all future calculations, we therefore assume that 21% of the fiber is protein, which gives the following $\sqrt{n}$ values (in parentheses, rounded to the nearest whole number) for fibers with the given diameter: 93.5 nm (9), 114.3 nm (11), 124.6 nm (12), 145.4 nm (14), 176.6 nm (17), 197.3 nm (19), 218.1 nm (21), 280.4 nm (27).

Additionally, we assume that each protofibril in the fiber cross-section contains 6 pairs of binding sites, which we call “binding doublets”, to which fibrinolytic enzymes can bind. These 6 binding doublets represent the 6 chains on a protofibril (2 pairs each of *α*, *β*, and *γ* chains). Initially, at each protofibril cross-section, 1 of the 6 binding doublets is exposed and available for binding, while the other 5 are cryptic until exposed by plasmin (more on this in “Model reactions” section below). In the original Bannish et al. model [10, 11], if a binding doublet had tPA bound to one site and plasminogen bound to the other, then tPA could convert that plasminogen to plasmin. In the current model, we only include plasmin, which can bind to either site on a doublet. While doublets are not necessary in the current model since we do not need two enzymes to bind in close proximity to each other, we retain the doublets so that the model will be easily extendable in the future.

#### Model reactions

**Fig 2:**
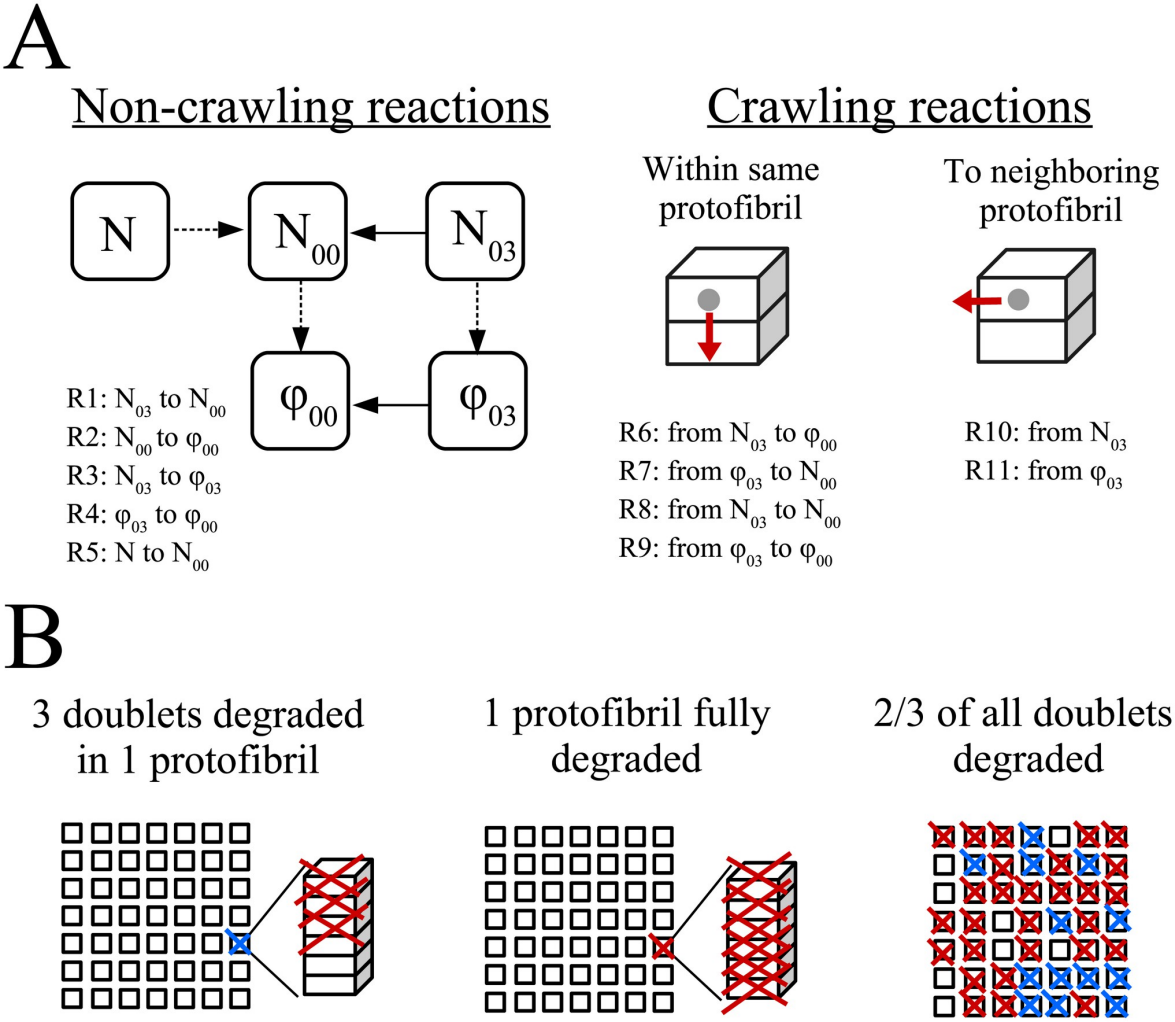
Model reactions. A: There are 11 reactions considered in the model: 5 involving unbinding, degradation, or exposure (non-crawling reactions), and 6 crawling reactions (4 within the same protofibril and 2 to a neighboring protofibril). *N* is a cryptic doublet, *N*_00_ is an exposed doublet with nothing bound, *N*_03_ is an exposed doublet with a plasmin bound, *ϕ*_00_ is a degraded doublet with nothing bound, and *ϕ*_03_ is a degraded doublet with a plasmin bound. Solid black arrows indicate an unbinding reaction, dashed arrows indicate a degradation or exposure reaction, and red arrows indicate a crawling reaction. Reactions are labeled as ‘R*i*’ with *i* = 1, …, 11. The unbinding reactions are R1 (plasmin unbinds from *N*_03_ resulting in *N*_00_) and R4 (plasmin unbinds from *ϕ*_03_ resulting in *ϕ*_00_). The exposure reaction is R5 (cryptic doublet *N* is exposed by plasmin to create *N*_00_). The degradation reactions are R2 (plasmin degrades *N*_00_, turning it into *ϕ*_00_) and R3 (plasmin degrades *N*_03_, turning it into *ϕ*_03_). There are 4 possible crawling reactions within the same protofibril: R6 (plasmin crawls from *N*_03_ to *ϕ*_00_, resulting in *N*_00_ and *ϕ*_03_), R7 (plasmin crawls from *ϕ*_03_ to *N*_00_, resulting in *ϕ*_00_ and *N*_03_), R8 (plasmin crawls from *N*_03_ to *N*_00_, resulting in *N*_00_ and *N*_03_), and R9 (plasmin crawls from *ϕ*_03_ to *ϕ*_00_ resulting in *ϕ*_00_ and *ϕ*_03_). Note that R8 and R9 do not change the state of the system, but do take time to occur, so we include them in the Gillespie algorithm. Finally, there are 2 possible crawling reactions from the given protofibril to a neighboring protofibril: R10 (plasmin crawls from *N*_03_ at this protofibril to a randomly chosen exposed doublet on a neighboring protofibril) and R11 (plasmin crawls from *ϕ*_03_ at this protofibril to a randomly chosen exposed doublet on a neighboring protofibril). B: The degradation reaction results in loss of doublets (degraded doublets represented by a red X in the leftmost figure). If some but not all of the doublets (protofibril chains) are degraded, then that protofibril is considered to be partially degraded (blue X). If all 6 doublets are degraded, then that protofibril is considered to be fully degraded (red X in middle figure cross-section). At a given fraction of degraded doublets, say 2/3, there will be a mix of undegraded, partially degraded, and fully degraded protofibrils (rightmost figure).

The model is initialized with a single plasmin molecule randomly placed on a protofibril on the outer edge of the fiber. This mimics the binding of plasmin to a fiber. In the current version of the model, we consider the situation in which only one plasmin molecule is present in a given fiber cross-section. Plasmin may be bound in several places along the length of a fiber, but we assume that it is unlikely for more than one plasmin molecule to bind to the same cross-section. This allows us to study the effect of a single plasmin molecule on the degradation of fibrin. We model 11 different reactions, which can be grouped into four main reaction types: exposure of cryptic doublets by plasmin, unbinding of plasmin from doublets, degradation of doublets by plasmin, and crawling of plasmin (Fig 2). Only exposed doublets are available for plasmin binding or crawling, hence the exposure reaction is requisite for the others to happen. Plasmin unbinding refers to the kinetic reaction where plasmin separates from the doublet to which it was bound. We assume that unbound plasmin is immediately inhibited by *α*2-AP, so once plasmin unbinds from the cross-section, degradation in that cross-section stops. The plasmin molecule can degrade any of the exposed doublets at its current protofibril cross-section. Finally, plasmin can crawl to other exposed doublets on the same protofibril or on neighboring protofibrils. We assume that crawling plasmin is protected from *α*2-AP inhibition. We allow plasmin to crawl on to degraded doublets (because we assume that there are frayed ends of protofibrils on either side of the cut created by plasmin when it chews through a protofibril), but plasmin cannot degrade the doublet further.

#### Model parameters

The four parameters in the model are the unbinding rate of plasmin from fibrin (*k*_unbind_), the crawling rate of plasmin between protofibrils (*k*_crawl_), the plasmin-mediated rate of fibrin degradation (*k*_deg_), and the plasmin-mediated rate of exposure of cryptic doublets (*k*_exp_). Baseline parameter values are taken from the literature (Table 1). However, since most rates needed for the model have not been directly measured, we run the model using a range of parameter values. Our process is to fix three parameters at their baseline values, then systematically change the fourth over a range of values.

**Table 1:** Baseline parameter values.

Parameter	Description	Value (1/s)	Reference
*k*_unbind_	kinetic unbinding rate of plasmin	0.05	[3]
*k*_crawl_	crawling rate of plasmin	57.6	[25]
*k*_deg_	plasmin-mediated rate of fibrin degradation	5	[26]
*k*_exp_	plasmin-mediated rate of cryptic doublet exposure	5	[11]

For this study, we always kept the degradation and exposure rates the same. Most of the references are to modeling papers, as it difficult to measure these rates experimentally. The crawling rate is taken to be the unbinding rate calculated in [25]. It was shown in [11] that in order for crawling to actually occur, *k*_crawl_ > > *k*_unbind_, which is why we take the larger estimate of plasmin unbinding rate from [25] as the crawling rate and the smaller estimate from [3] as the unbinding rate.

#### Model simulations and statistical analysis

The model is numerically simulated using custom MATLAB code that employs the Gillespie algorithm, which generates a statistically exact solution of the Master equation describing the change in probability of the system at a particular state at a given time [27, 28]. The Gillespie algorithm randomly determines the next reaction that will occur and the time at which the next reaction will happen. The master equation for our model is

$$\begin{array}{c} \frac{\partial P}{\partial t}\left(\vec{x},t|{\vec{x}}_{0},t_{0}\right)=\sum_{j=1}^{11}(a_{j}\left(\vec{x}-{\vec{v}}_{j}\right)P\left(\vec{x}-{\vec{v}}_{j},t|{\vec{x}}_{0},t_{0}\right)-a_{j}\left(\vec{x}\right)P\left(\vec{x},t|{\vec{x}}_{0},t_{0}\right)) \end{array}$$ (2)

where $P(\vec{x},t|{\vec{x}}_{0},t_{0})$ is the probability of the system being in state $\vec{x}$ at time *t* given that the system was in state ${\vec{x}}_{0}$ at time *t*_0_. The state vector, $\vec{x}$, is defined as

$$\begin{array}{c} \vec{x}=\left[N_{00},N_{03},\phi_{00},\phi_{03},N\right] \end{array}$$ (3)

using the notation from Fig 2, where *x*_*i*_ is the number of doublets of type *i* in the system. The stoichiometric matrix describing the 11 possible reactions that can occur is

$$\begin{array}{c} v=[\begin{array}{c} {\vec{v}}_{1}\\ {\vec{v}}_{2}\\ {\vec{v}}_{3}\\ {\vec{v}}_{4}\\ {\vec{v}}_{5}\\ {\vec{v}}_{6}\\ {\vec{v}}_{7}\\ {\vec{v}}_{8}\\ {\vec{v}}_{9}\\ {\vec{v}}_{10}\\ {\vec{v}}_{11} \end{array}]=[\begin{array}{ccccc} +1 & -1 & 0 & 0 & 0\\ -1 & 0 & +1 & 0 & 0\\ 0 & -1 & 0 & +1 & 0\\ 0 & 0 & +1 & -1 & 0\\ +1 & 0 & 0 & 0 & -1\\ +1 & -1 & -1 & +1 & 0\\ -1 & +1 & +1 & -1 & 0\\ 0 & 0 & 0 & 0 & 0\\ 0 & 0 & 0 & 0 & 0\\ +1 & -1 & 0 & 0 & 0\\ 0 & 0 & +1 & -1 & 0 \end{array}] \end{array}$$ (4)

and the propensity function is

$$\begin{array}{c} \begin{array}{cc} a & =[k_{\text{unbind}}x_{2},k_{\text{deg}}x_{1}n,k_{\text{deg}}x_{2}n,k_{\text{unbind}}x_{3},k_{\text{exp}}x_{5}n,k_{\text{crawl}}x_{2}x_{3},\\ & k_{\text{crawl}}x_{1}x_{4},k_{\text{crawl}}x_{4}x_{3},k_{\text{crawl}}x_{2}x_{1},k_{\text{crawl}}x_{2},k_{\text{crawl}}x_{4}] \end{array} \end{array}$$ (5)

where *n* is 0 if there is no plasmin at the given protofibril and 1 if there is a plasmin molecule at the given protofibril. Note that the two rows of zeroes corresponding to reactions 8 and 9 represent reactions that do not change the state of the system, but do take time: plasmin crawling from *N*_00_ to *N*_03_ on the same protofibril and plasmin crawling from *ϕ*_03_ to *ϕ*_00_ on the same protofibril. A description of all reactions is provided in the Fig 2 caption.

**Fig 3:**
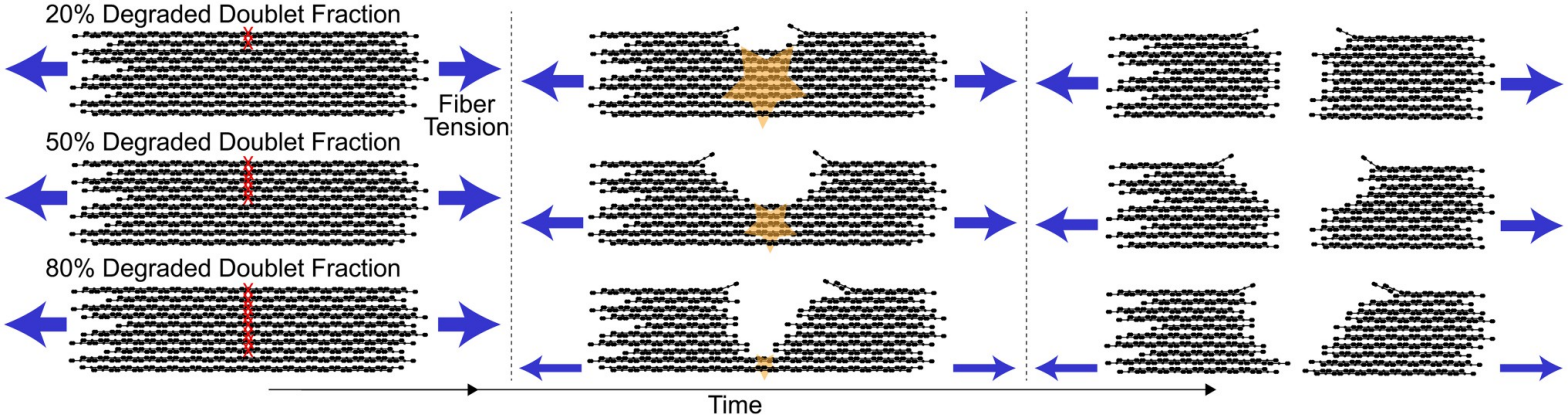
Cartoon of different degraded doublet fractions and the effect of tension. Since fibers are under tension, we assume that they cleave into two pieces before all the fibrin (doublets) in a cross-section is degraded by plasmin. In the top figure, only 20% of the doublets need to be degraded (red X’s), before tension causes the fiber to snap (orange star). In the middle figure, 50% of the doublets need to be degraded before tension causes the fiber to snap. In the bottom figure, 80% of the doublets need to be degraded before tension causes the fiber to snap. Blue arrows indicate the direction of force acting on the fiber, and their thickness scales with the magnitude of the force. Time increases from left to right.

The fiber is considered to be cleaved when 2/3 of the binding doublets within the cross-section have been degraded [10, 11]. This assumption accounts for the fact that fibers are under tension, so after some threshold amount of degradation, the fiber snaps [14]. We use 2/3 as the baseline value, but we also investigate other degradation fractions (Fig 3). “Cleavage time” is defined in this study to be the time at which the assigned fraction of binding doublets have degraded.

Since the model is stochastic, we run the Gillespie algorithm 10,000 independent times to generate enough data to report on the statistics. We choose 10,000 simulations to ensure that even with the most extreme parameter values tested, at least 500 of the simulations result in successful cleavage of the fiber. We save the degradation state of each doublet at each protofibril every time step, the location of the plasmin molecule at each time step, and the cleavage time. We compute the median, 5th, and 95th percentiles of the cleavage time data. To fit lines to data we use least squares regression.

For spatial autocorrelation analysis we use the “moran.test” function in the R package “spdep” to perform a Moran’s I test [29]. The spatial weights matrix contains 1’s and 0’s, depending on whether a protofibril is a direct neighbor of a given protofibril or not, respectively. It is the same matrix that is used in the model to determine which protofibrils a specific plasmin molecule can crawl to. Additional details of the Moran’s I test and analysis are in the Supporting Information S1 Text.

### Laboratory experiments

The lysis of individual fibrin fibers was analyzed from stationary clots formed on microscopic ridges as described previously [14]. Briefly, clots were created by combining fibrinogen (1 mg/mL; Enzyme Research Labs, South Bend, IN), thrombin (0.1 U/mL; Enzyme Research Labs, South Bend, IN), and Alexa-488 labeled fibrinogen (0.015 mg/mL; Invitrogen) at the listed, final concentrations and allowed to polymerize at 37°C in a humid environment. After incubating for an hour, the majority of the clot was removed through gentle pipetting, and the remaining isolated fibers were imaged. A Leica DMi8 epifluorescent microscope was used to image digestion caused by the addition of 0.066 U/mL of plasmin. Timelapse images were taken during the digestion, and the cleavage time for each fiber was recorded, as described previously [14]. Image timelapses were imported into ImageJ and the apparent diameter of each fiber was measured 5 times, as follows. Fluorescent intensity was measured for perpendicular fiber cross-sections at five locations along each fiber using the Plot Profile tool in ImageJ. Intensity profiles were fit with a Gaussian curve after subtracting out the background fluorescence. The apparent fiber diameter was estimated as the standard deviation of the Gaussian curve times four, which includes 95.4% of the fluorescent intensity of the fiber and allows for a consistent metric of diameter for differing fibers. The diameter measurements at the five different locations were averaged, and the average was correlated with the corresponding cleavage time for each fiber. Because fibers are diffraction limited (diameter ∼100–200 nm) [30] the diameters measured in this way do not represent true diameters but can be used to compare fibers to each other, thus we refer to this as the “apparent diameter” of the fiber. To facilitate a comparison between fibers, fiber diameters were scaled by dividing each apparent diameter by the apparent diameter of the second-smallest fiber. The smallest fiber had an apparent diameter that was 40% smaller than the next-smallest fiber, suggesting that it was statistically much thinner than any of the fibers measured. Scaled diameters were graphed against the corresponding cleavage times.

## Results

One of the primary objectives of this research was to develop a mathematical model whose lysis times and digestion mechanisms were congruous with those observed experimentally for single fibrin fibers. However, many of the rate constants governing these processes are unknown, or have conflicting values in the literature (for example, literature estimates for *k*_deg_ range from about 0.02 − 25 s^−1^ [2, 31, 32]). Thus, our approach was to investigate how the model behaves under various biochemical conditions, and then propose reasonable parameter ranges based on comparison of the model data to experimental data.

### Cleavage time and success rate

**Fig 4:**
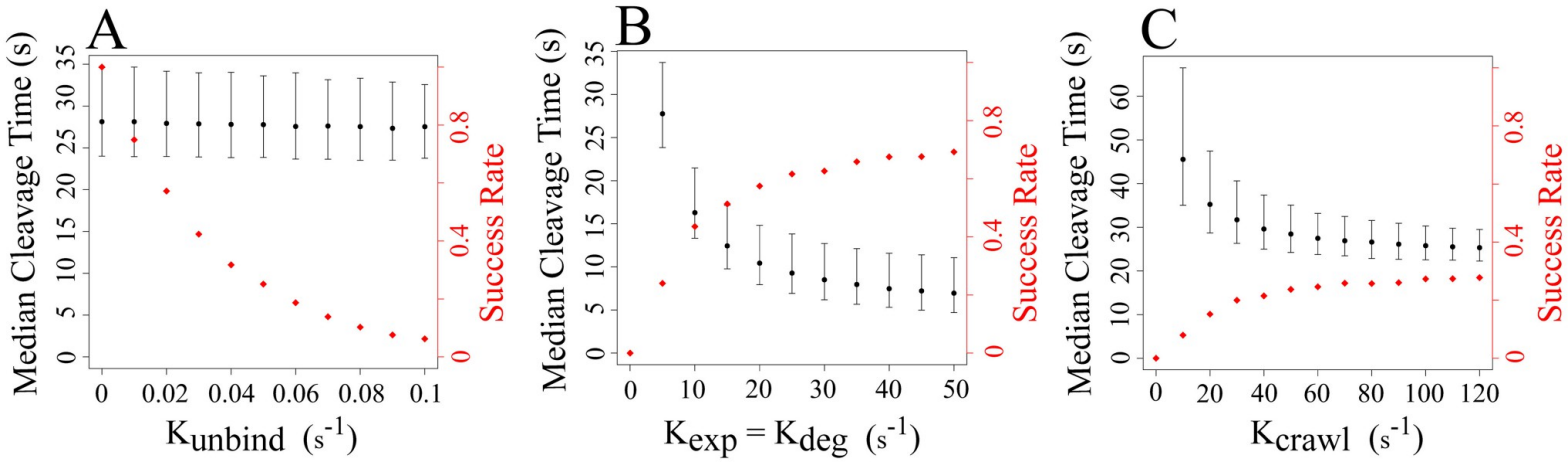
Median fiber cleavage time (black) and the fraction of successful runs (red) for different parameter values. A: Median cleavage time and cleavage success rate as a function of the kinetic unbinding rate of plasmin from fibrin, *k*_unbind_. B: Median cleavage time and cleavage success rate as a function of the degradation and exposure rates of fibrin by plasmin, *k*_deg_ and *k*_exp_, respectively. C: Median cleavage time and cleavage success rate as a function of the crawling rate of plasmin, *k*_crawl_. The top and bottom error bars show the 95th and 5th percentiles, respectively.

We began by running the model with the baseline parameters from Table 1 and found that the median lysis time was 27.76 s, and 25.11% of runs resulted in cleavage (Fig 4). A run fails to result in cleavage of the fiber if the plasmin molecule unbinds from the cross-section before 2/3 of the binding doublets have been degraded. We refer to runs that result in cleavage of the fiber as “successful runs”, and to the fraction of successful runs out of 10,000 as the “success rate”. Next, we systematically varied either the unbinding rate (Fig 4A), exposure and degradation rates (which we kept equal for the present study, Fig 4B), or the crawling rate (Fig 4C) while keeping all other parameters at their baseline values.

Changing the unbinding rate from 0 s^−1^ to 0.1 s^−1^ had a negligible effect on median cleavage time but reduced the success rate from 100% to 6.23% (Fig 4A). The success rate data make sense given that if plasmin never unbinds (*k*_unbind_ = 0 s^−1^), then every run results in cleavage; however, as the plasmin unbinding rate increases, plasmin is more likely to unbind from the cross-section before cleavage occurs. The median cleavage times were not very different because they were a result of the runs in which plasmin stayed bound long enough to degrade 2/3 of the binding doublets. Since the crawling, exposure, and degradation rates were the same in all of these runs, the resulting cleavage times were also very similar.

Varying the exposure and degradation rates from 5 s^−1^ to 50 s^−1^ noticeably decreased the median cleavage time from 27.76 s to 6.95 s (Fig 4B). Clearly, as the rate at which plasmin exposes and degrades binding doublets increases, it takes less time for 2/3 of the binding doublets to be degraded. Similarly, low exposure and degradation rates resulted in very low success rates (24.01%, for *k*_exp_ = 5 s^−1^), while higher rates resulted in up to 69.24% successful runs. This is because if plasmin is slow to degrade binding doublets, then there is an increased chance that it will unbind before cleavage occurs. Interestingly, median cleavage times and cleavage success rates eventually leveled out with increasing exposure and degradation rate. This limiting behavior was due to the unbinding and crawling parameters, which prevented the fibers from being degraded even faster.

As the plasmin crawling rate was increased from 10 s^−1^ to 120 s^−1^, the median cleavage time decreased from 45.53 s to 25.32 s, and the success rate increased from 7.94% to 27.75% (Fig 4C). Higher crawling rates mean that plasmin moves within and between protofibrils more quickly, therefore has access to more binding doublets that it can expose or degrade, and hence, it takes less time for 2/3 of the binding doublets to be degraded. The success rate never got higher than 28%, and did not vary much over a wide range of crawling rates (30 s^−1^ ≤ *k*_crawl_ ≤ 120 s^−1^), indicating that crawling rate likely has only a small effect on cleavage success rate. Both the median cleavage time and the success rate leveled out with increasing crawling rate, suggesting that the unbinding, exposure, and degradation rates were limiting factors.

### Degradation pattern of lysis

**Fig 5:**
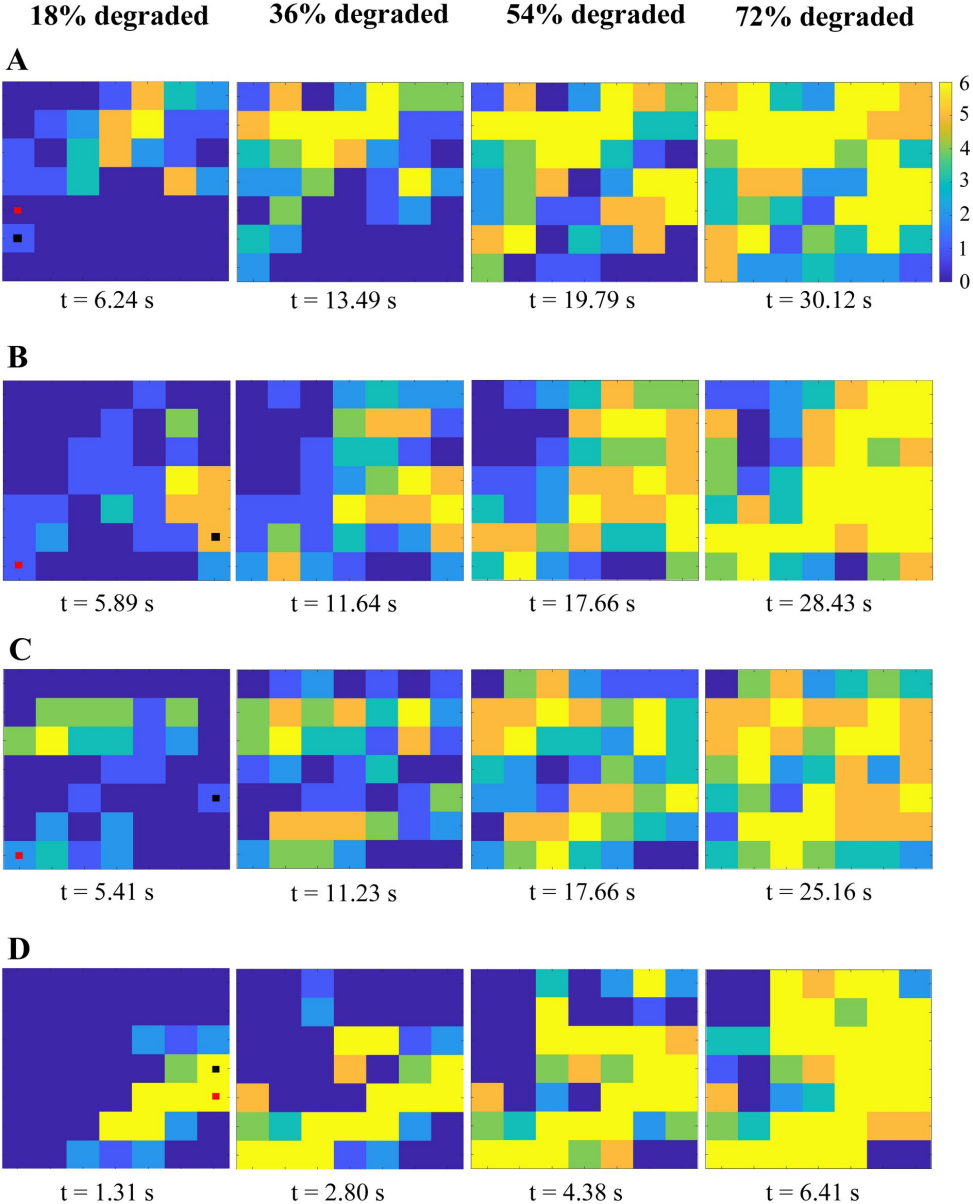
Degradation pattern for four different parameter sets. Snapshots were taken when the percentage of degraded doublets in the cross-section was 18%, 36%, 54%, and 72%, from left to right. Each pixel represents a protofibril. The color bar indicates the number of degraded doublets at the given protofibril from 0 (blue) to 6 (yellow). The small red square shows the initial location of plasmin and the small black square shows where degradation first occurred. A: Baseline parameter values. B: Lower unbinding rate (*k*_unbind_ = 0.01 s^−1^), other parameters at baseline values. C: Higher crawling rate (*k*_crawl_ = 120 s^−1^), other parameters at baseline values. D: Higher degradation and exposure rates (*k*_deg_ = *k*_exp_ = 45 s^−1^), other parameters at baseline values.

We visualized the degradation of binding doublets within a given fiber cross-section to determine if the pattern of lysis was affected by different reaction rates (Fig 5). Since the model is stochastic, results differ for each of the 10,000 independent simulations. Hence, we chose to display a representative simulation that had a cleavage time corresponding to the median cleavage time for the given set of parameters. We considered a simulation using baseline parameters (Fig 5A), and three additional simulations that all differed from baseline in just one parameter: lower unbinding rate, *k*_unbind_ = 0.01 s^−1^ (Fig 5B); higher crawling rate, *k*_crawl_ = 120 s^−1^ (Fig 5C); and higher exposure and degradation rate, *k*_deg_ = *k*_exp_ = 45 s^−1^ (Fig 5D). For each set of parameters considered, we plotted the number of degraded binding doublets at each protofibril in the cross-section at four stages of lysis: 18%, 36%, 54%, and 72% of total binding doublets degraded.

The pattern of lysis was discernibly different only for the higher exposure and degradation rate case (Fig 5D). Since plasmin exposed and degraded binding doublets more quickly, degradation was more localized nearby to where plasmin was initially placed. In the other 3 cases, plasmin crawling resulted in a more diffuse pattern of degradation throughout the cross-section. We can see this by comparing the number of fully degraded protofibrils (yellow pixels) in each cross-section at the different percentages of total degradation. For example, the baseline, lower unbinding rate, and higher crawling rate simulations have 7, 3, and 2 fully degraded protofibrils at 36% degradation, compared to 12 in the higher exposure and degradation rate simulation. At 72% degradation, those numbers are 19, 21, and 12 compared to 26. Expectedly, cleavage was by far the fastest in the higher exposure and degradation simulation, and similar in the remaining three simulations.

A spatial autocorrelation analysis confirmed that the degradation pattern was more localized in the higher exposure and degradation rate case compared to the other three parameter sets (Supporting Information S1 Text). Running a Moran’s I test on the 54% degraded data obtained from 1000 simulations of each parameter set resulted in a larger median correlation coefficient for the higher exposure and degradation rate scenario (0.570) than the baseline (0.458), lower unbinding rate (0.472), and higher crawling rate (0.432) scenarios.

### Fraction of degraded doublets for cleavage

**Fig 6:**
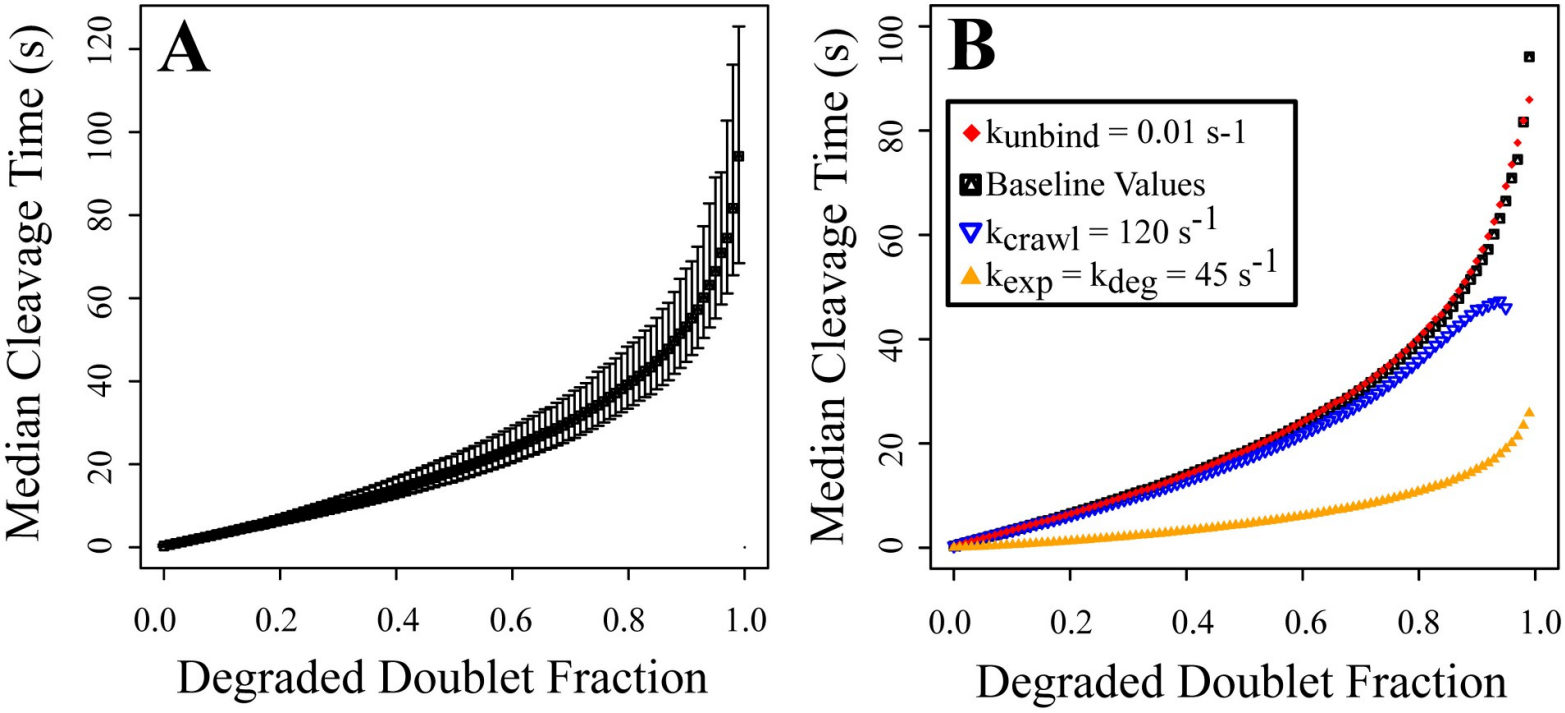
Median fiber cleavage time at different degraded doublet fractions. The results are obtained from 10,000 simulations. A: Baseline parameter values. The top and bottom error bars show the 95th and 5th percentiles, respectively. B: Different parameter values. All parameter values are held fixed at the values listed in Table 1 except for the following: *k*_unbind_ = 0.01 s^−1^ (red diamond), *k*_krawl_ = 120 s^−1^ (empty blue triangle), *k*_exp_ = *k*_deg_ = 45 s^−1^ (solid orange triangle).

For the results discussed so far, we assumed that the fiber was cleaved once 2/3 of the binding doublets in the cross-section were degraded. This assumption was meant to account for the fact that fibers polymerize with inherent tension, and therefore often snap into two segments once enough of the fibrin within a cross-section has been degraded (Fig 3). The 2/3 fraction was used in earlier models [10, 11, 33], but the physiological fraction of fibrin in a fiber cross-section that must be degraded before the fiber snaps is unknown. Thus, we used the model to investigate how median cleavage time was affected by the fraction of binding doublets that need to be degraded prior to tension taking over and pulling the fiber apart into two segments (Fig 6).

We ran the model 10,000 independent times for each parameter set considered and saved the time at which each fraction from 0.01, 0.02, … 0.99, 1 of binding doublets were degraded. Fig 6A shows the resulting data for the baseline parameters. The median cleavage time increased nonlinearly with the fraction of degraded binding doublets needed for cleavage. The error bars, representing the 5th and 95th percentiles, were much larger for higher fractions of degradation, indicating that there was more variability in the data. It should be noted that the success rate was much lower as the degraded doublet fraction increased (Fig A in S1 Text).

Similar results were observed for the higher crawling rate, higher exposure and degradation rate, and lower unbinding rate (Fig 6B). The lower unbinding rate data was very similar to the baseline data, which makes sense given that unbinding rate only affected the success rate, not the median cleavage time (Fig 4A). Likewise, the higher crawling rate data was very similar to the baseline data until a degraded doublet fraction of about 0.8. Above this fraction of degraded binding doublets needed for cleavage, median cleavage time was shorter in the higher crawling rate case than in the baseline case. This is likely an effect of the lower lysis success rate (Fig 4C) in the higher crawling rate case. Fewer runs made it as far as having ≥ 0.8 of the doublets degraded (in fact, no runs made it to > 0.95 degraded), so fewer data points were used in the median calculation. That means individual fast runs could have a greater impact on the median cleavage time. The fastest median cleavage times occurred in the higher exposure and degradation rate case. The shape of the median cleavage time curve was similar to the baseline curve, but the median cleavage times at each fraction of degraded binding doublets were much lower. Median cleavage curves with error bars for the non-baseline parameter cases can be found in Supporting Information S1 Text (Fig B).

### Effect of fiber diameter on cleavage time

**Fig 7:**
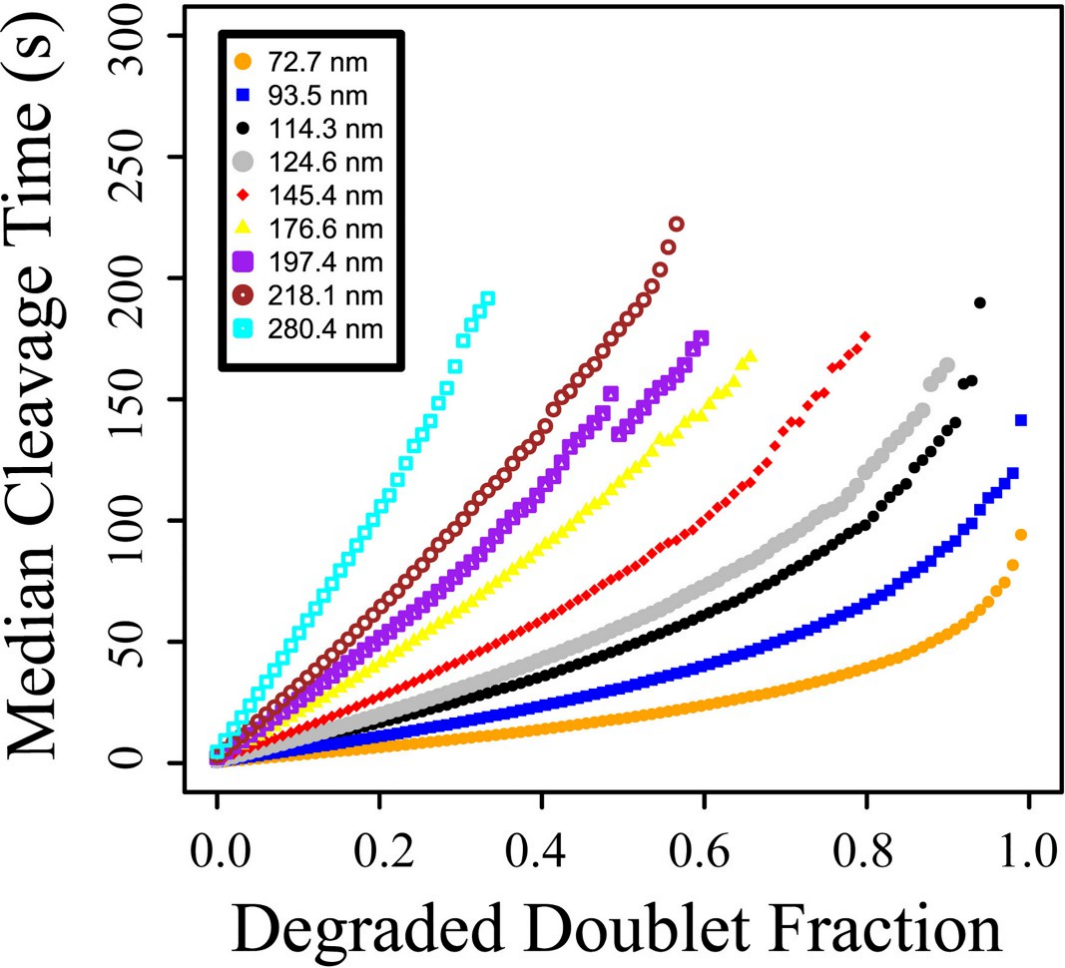
Median cleavage time of different fiber sizes at different degradation fractions. The median cleavage times were measured with rate constants at their baseline values. The maximum degraded doublet fraction reached in the simulations decreases with thicker fibers because plasmin leaves the cross-section before it can degrade more doublets.

To understand how single fiber cleavage time is affected by fiber diameter, we repeated the fraction of degraded doublets for cleavage simulations for 8 additional fiber diameters (Fig 7). As the fiber diameter increased, the median cleavage time also increased at each fraction of degraded doublets tested. Because thicker fibers took longer to cleave, plasmin unbound from the fiber cross-sections before 100% (and sometimes before as little as 35%) of the doublets in the cross-section had been degraded. For instance, in the 280.4-nm diameter fiber case, in all 10,000 independent simulations plasmin unbound before 36% of the doublets had been degraded.

### Comparison of experimental and model results

**Fig 8:**
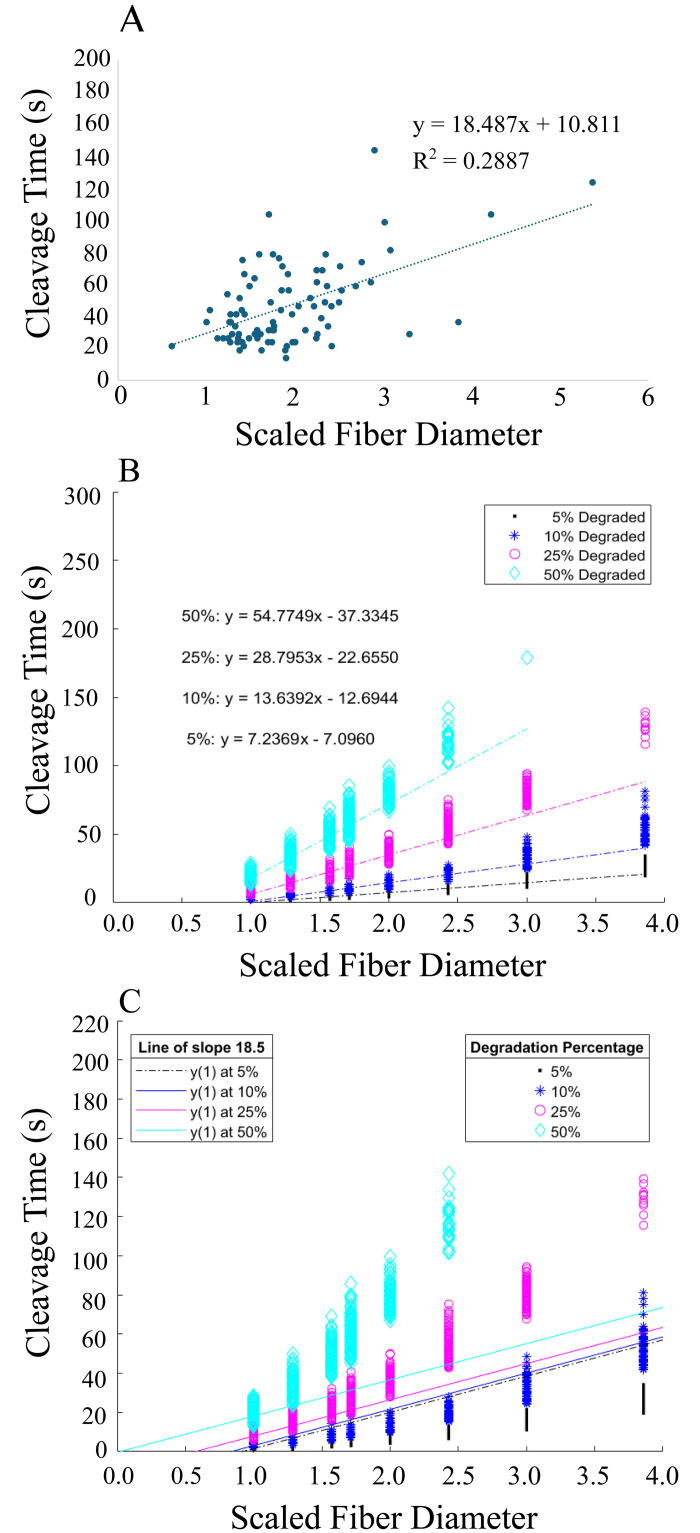
Cleavage time as a function of scaled fiber diameter. A: Experimental data showing the cleavage time as a function of the scaled fiber diameter. The line of best fit had slope 18.487. B: Model data with baseline parameters showing cleavage time as a function of the scaled fiber diameter. cleavage was defined as when 0.05 (black, 5%), 0.10 (blue, 10%), 0.25 (pink, 25%), or 0.5 (cyan, 50%) of the doublets in the cross-section had been degraded. Lines of best fit were computed for each of those four different sets of data. C: The same model data as in B, but with lines of slope 18.5 passing through the median cleavage time at scaled fiber diameter 1.0.

Laboratory experiments can measure the cleavage time of fibers of different diameters, but it is not currently possible to measure the fraction of fibrin within a fiber cross-section that has been degraded when cleavage of the fiber occurs. Thus, we combined our modeling results with an experimental study to identify how much fibrin must be degraded for a fiber to snap. The experimental data indicated that cleavage time increased with increasing scaled fiber diameter, and the best-fit line to the data had slope 18.5 (Fig 8A). Since it is unknown what fraction of fibrin in the cross-section was degraded at the time of experimental cleavage, we collected model data at 4 different fractions: 0.05, 0.10, 0.25, and 0.50. Fig 8B shows the cleavage time data and lines of best fit for baseline parameters (similar results for higher exposure and degradation rate data are provided in (Fig C in S1 Text)). To obtain a similar slope to the experimental data, we needed only between 0.1 and 0.25 of binding doublets in a fiber cross-section to be degraded for cleavage to occur. However, this assumed that the fraction of degraded doublets needed for fiber cleavage was the same, regardless of fiber diameter. Using the same data as Fig 8B, we plotted lines with slope 18.5 (to match the experimental slope), passing through the corresponding median cleavage time at scaled fiber diameter 1.0 (Fig 8C). For example, the median cleavage time at scaled fiber diameter 1.0 is 18.13 s for the 50% degradation data, so the cyan line of slope 18.5 goes through the point (1, 18.13). Here we saw that thinner fibers required a higher fraction of doublets to be degraded compared to thicker fibers. For example, with baseline parameters and using the 50% degradation data, we matched the experimental data if 0.5 of the doublets were degraded in the thinnest fiber cross-section, 0.25 were degraded in the fiber with twice the diameter, and 0.1 were degraded in the fiber with 4-times the diameter (Fig 8C).

## Discussion

We created a 2-dimensional stochastic model to study cleavage of single fibrin fibers. Previous models of fibrinolysis mainly considered network-level degradation of clots exposed to tPA [2, 5, 9, 11, 34]. In the present study, we aimed to identify how the rates and patterns of degradation of a single fibrin fiber were affected by the movement, unbinding, and degradation rates of individual plasmin molecules. We used plasmin rather than tPA to initiate lysis in order to best match single-fiber lysis experiments, many of which use plasmin [14, 20, 22, 35, 36].

Models can be useful for identifying mechanisms underlying biological processes, but often the parameter values needed for the models are unknown. For instance, the literature contains a range of dissociation constants for plasminogen (and by association, plasmin) binding to fibrin [37–39]. Measuring individual binding and unbinding rates is more difficult, and to our knowledge there is only one estimate of an unbinding rate of plasmin [25]. Likewise, it is difficult to measure with certainty the plasmin-mediated rates of exposure of cryptic binding sites and of degradation of fibrin. Therefore, we used best-guess values as our baseline parameters and then systematically varied individual rates to see how they affected single fiber cleavage. We found that the unbinding rate of plasmin from fibrin (*k*_unbind_) had virtually no effect on the median cleavage time, but as *k*_unbind_ increased, the cleavage success rate dropped precipitously (Fig 4A). So, the plasmin unbinding rate determines how likely a fibrin fiber exposed to a single plasmin molecule is to degrade, but not how fast that fiber degrades. Increasing exposure and degradation rates (*k*_exp_, *k*_deg_) and crawling rates (*k*_crawl_) all resulted in lower median cleavage times and higher cleavage success rates (Fig 4B and 4C). However, the cleavage times and success rates seemed to level out, indicating that increasing the rate constants above a certain value (namely, *k*_exp_ = *k*_deg_ ≈ 35 s^−1^ and *k*_crawl_ ≈ 60 s^−1^) has little effect on cleavage. This knowledge is helpful for future modeling efforts, which can test a narrower range of parameter values.

Additionally, we can compare the model and experimental cleavage times to narrow the possible parameter ranges even further. Most of the experimental fibers cleaved in 15–80 s (Fig 8A). To get cleavage times in that range with the model, we need *k*_exp_ = *k*_deg_ ≤ 10 s^−1^. Any value of *k*_crawl_ or *k*_unbind_ in the ranges we tested produces the desired cleavage times. We conclude that the rate of plasmin-mediated exposure of new binding sites and of fibrin degradation is likely less than 10 s^−1^. Future experimental work investigating the fibrinolytic effects of single plasmin molecules could provide an estimate of the cleavage success rate, which would allow us to estimate a range of plausible plasmin unbinding rates. Since it is not known for certain if plasmin crawls across fibers, the *k*_crawl_ parameter is harder to test experimentally. However, our modeling work shows that crawling rates from 10 − 120 s^−1^ all result in experimentally-plausible cleavage times. So, as long as plasmin *does* crawl (*k*_crawl_ ≠ 0 s^−1^), the particular value of the crawling rate is not as important, though future modeling efforts could investigate how single fiber cleavage is affected if plasmin molecules encounter steric hindrance as they crawl through the fiber. If it turns out that plasmin does not crawl along fibrin, then the model would need to be adjusted so that multiple plasmin molecules could bind to doublets within the fiber cross-section; otherwise, there would be no way for plasmin to degrade fibrin at locations away from where it initially bound. We made the assumption that when plasmin unbinds from the fiber cross-section, it diffuses away from the current cross-section, and hence we do not consider diffusion of plasmin *within* a given cross-section. This assumption is based on a calculation for the probability of a tPA molecule rebinding to the same cross-section, which was shown to be on the order of 10^−6^ [10].

The pattern of degradation within a fiber cross-section was only discernibly different at high exposure and degradation rates (Fig 5, Table A in S1 Text), but since comparison with experiments suggests that these rates should not be greater than 10 s^−1^, we conclude that the pattern of degradation within a fibrin fiber is similar under a wide range of physiologically realistic parameter values. Likewise, the fraction of fibrin in a fiber cross-section that must be degraded before the fiber is cleaved is only appreciably different at high exposure and degradation rates (Fig 6B). For the model fiber with diameter 72.7 nm, degraded doublet fractions of ∼ 0.4 − 1.0 give cleavage times in the range of experimental values. As fiber diameter increases, a smaller fraction of doublets need to be degraded to achieve a given cleavage time (Fig 7). For example, just 0.2 of the doublets in the cross-section of a 197.4-nm-diameter fiber need to be degraded to get a median cleavage time of about 50 s, compared to over 0.9 of the doublets in a 72.2-nm-diameter fiber. Because fibrin fiber diameter values approach or fall below the diffraction limit, it is currently challenging to design experiments that accurately measure diameter values while simultaneously measuring a time series of plasmin digestion [30]. Thus, in our experimental lysis studies, we estimated fiber diameters based off fluorescence intensity, and then used the scaled diameter to compare how this property relates with cleavage times (Fig 8). In the future, it may be possible to use time resolved super-resolution microscopy techniques to compare more directly experimental diameters with the model, however those techniques are not yet commonly available. This would have a two-fold benefit: 1) by comparing the model and experimental results as in Fig 8, we could obtain a more accurate estimate for how much fibrin in a fiber cross-section needs to be degraded before the fiber snaps; and 2) we could identify which parameter sets give median cleavage times most like experimental cleavage times, and therefore propose tighter ranges for some of the unknown rate constants.

The line of best fit to the experimental cleavage time vs scaled fiber diameter data has a slope of 18.5 (Fig 8A). With baseline model parameters, to match the experimental slope it would take only between 0.1 and 0.25 of the doublets in the fiber cross-section to be degraded for the fiber to be cleaved (Fig 8B). This seems surprisingly low, so even though our cleavage time data suggests that the exposure and degradation rates are likely ≤ 10 s^−1^, we tested a higher rate of 45 s^−1^ to get a fuller understanding of the interplay between fiber diameter, fraction of fibrin degraded, and cleavage time (Fig C in S1 Text). At the higher exposure and degradation rate, 0.5 of the doublets need to be degraded to match the experimental slope. This makes sense: to reach a given cleavage time, if the rate of degradation is faster, then more of the fibrin must be degraded. These modeling results were based on the assumption that all fibers require the same fraction of doublets to be degraded for fiber cleavage. However, thicker fibers are likely under more tension than thinner fibers due to protofibril packing [40], so it is possible that less of the fibrin in the thicker fiber cross-section must be degraded before tension takes over and the fiber snaps. To test this, we fit several lines with slope 18.5 to the model data and looked at where they intersected the different fraction of doublets data (Fig 8C, Fig C in S1 Text). In all cases, at smaller fiber diameters the lines intersected higher degraded doublet fractions than at larger fiber diameters. Thus, we propose that for single fiber cleavage, thinner fibers require a higher fraction of fibrin in the cross-section to be degraded compared to thicker fibers, consistent with the idea that thicker fibers are under more tension. The degradation fractions we identified (ranging from 0.1–0.5) are lower than we expected, but until a careful experimental assessment of the effects of true fiber diameter on cleavage rate is conducted, they are the best estimates available. Identifying what fraction of fibrin within a fiber cross-section must be degraded before the fiber is cleaved will further our understanding of the role of tension in fibrinolysis.

The model presented here is specifically for plasmin-induced fibrinolysis, but was created in such a way as to be easily generalized. For example, future versions of the model could investigate situations in which more than one plasmin molecule is present in the fiber cross-section, or could include plasmin, plasminogen, and tPA as well as fibrinolytic inhibitors. These modifications would require additional reactions and states in the Gillespie algorithm, but the framework would be the same. Additionally, we plan to couple this single-fiber lysis model to a macroscale model of full clot lysis initiated by plasmin (similar to the Bannish, et al. model of tPA-initiated lysis [11]). While the model currently represents an uncrosslinked fiber in order to best match the experimental conditions, in the future it could be modified to study lysis of FXIIIa-crosslinked fibers by adjusting model parameters and including the direct crosslinking of the plasmin inhibitor *α*2-AP to the fibrin fiber. Since the crosslinked *α*2-AP would inhibit the crawling plasmin, single fiber lysis would be impeded. The proposed crosslinked fibrin model could then be used to investigate the affect of anti-*α*2-AP antibodies, which are currently in Phase II trials to help dissolve blood clots in patients with deep vein thrombosis [41].

In conclusion, the model presented here, coupled to experimental data, provides important information about plasmin-induced single fibrin fiber cleavage and serves as a framework for future modeling studies. With our combined modeling and experimental approach we were able to propose parameter ranges for unknown biological rate constants, estimate the fraction of fibrin within a fiber cross-section that must be degraded for the fiber to cleave in two, and propose that the fraction is higher in thinner fibers and lower in thicker fibers. This information is particularly important for the continued understanding of the role tension plays in fibrinolysis.

## Supporting information

S1 Text Supporting information PDF. PDF with all supporting information.
(PDF)
