# Supplementary material for: A mathematical model of plasmin-mediated fibrinolysis of single fibrin fibers
Source: PLoS Comput Biol. 2024 Dec 20;20(12):e1012684. doi: 10.1371/journal.pcbi.1012684 (PMC11703044; doi:10.1371/journal.pcbi.1012684)
Supplement: S1 Text — PDF with all supporting information. (PDF) [file pcbi.1012684.s001.pdf]

Supporting Information: A Mathematical Model of Plasmin-Mediated Fibrinolysis of Single Fibrin Fibers

Roukayatou R. Ouedraogo[1], Hannah K. Sowers[2], Spencer R. Lynch[2], Nathan E. Hudson[2], Brittany E. Bannish[1*]

1 Department of Mathematics and Statistics, University of Central Oklahoma, Edmond, Oklahoma, USA
2 Department of Physics, East Carolina University, Greenville, North Carolina, USA

* bbannish@uco.edu

Supporting information

To quantitatively test if the spatial degradation patterns (Fig 5) were different, we ran a Moran's I test. We ran 1000 independent model simulations with each parameter set and for the simulations that resulted in cleavage, we saved the number of degraded doublets on each protofibril at the times at which 54% and 72% of the total doublets were degraded. This was the data that we evaluated with the Moran's I test. Using the built-in function "moran.test" in the R package "spdep", we obtained the weighted correlation coefficient and p-value for each simulation. Results from this analysis are in Table A. The spatial weights matrix we used was determined as follows. We enumerated the protofibrils from 1 to 49. For a given protofibril, a weight of 1 was assigned to neighboring protofibrils (protofibrils that plasmin could crawl to in one step), and a weight of 0 was assigned to all other protofibrils, resulting in a 49×49 weight matrix of 0's and 1's. Thus, we tested whether the number of degraded doublets on a protofibril was similar to the number of degraded doublets on nearby protofibrils. The Moran's I correlation coefficient ranges from -1 (indicating perfect spatial clustering of dissimilar values) to 1 (indicating perfect clustering of similar values). Our positive correlation coefficients indicated that protofibrils near a given protofibril were likely to have similar numbers of degraded doublets. Since the $k_{\text{deg}} = k_{\text{exp}} = 45\ \text{s}^{-1}$ parameter set had the biggest median correlation coefficient, we concluded that the degradation pattern was more localized in this case than in the other 3 parameter sets tested.

	54% Degradation		72% Degradation	
Parameter Set	Median Correlation Coefficient (95% CI)	Runs	Median Correlation Coefficient (95% CI)	Runs
Baseline	0.458 (0.231, 0.702)	183	0.404 (0.209, 0.630)	179
$k_{\mathrm{unbind}} = 0.01\ \mathrm{s}^{-1}$	0.472 (0.217, 0.711)	687	0.417 (0.187, 0.662)	682
$k_{\mathrm{crawl}} = 120\ \mathrm{s}^{-1}$	0.432 (0.191, 0.691)	213	0.370 (0.180, 0.571)	212
$k_{\mathrm{deg}} = k_{\mathrm{exp}} = 45\ \mathrm{s}^{-1}$	0.570 (0.286, 0.766)	659	0.493 (0.240, 0.727)	654

Table A. Results of spatial autocorrelation test for fiber cross-sections with 54% and 72% of the total doublets degraded. For each parameter set, we list the median correlation coefficient followed by the 95% confidence interval. "Runs" indicates the number of independent simulations, out of 1000, for which cleavage occurred and the p-value for the correlation coefficient was < 0.05. There were 8 (from $k_{\mathrm{unbind}} = 0.01\ \mathrm{s}^{-1}$) and 7 (from $k_{\mathrm{crawl}} = 120\ \mathrm{s}^{-1}$) runs that needed to be excluded from the 54% degradation data due to high p-values, and 4 (from baseline), 13 (from $k_{\mathrm{unbind}} = 0.01\ \mathrm{s}^{-1}$), 8 (from $k_{\mathrm{crawl}} = 120\ \mathrm{s}^{-1}$), and 5 (from $k_{\mathrm{deg}} = k_{\mathrm{exp}} = 45\ \mathrm{s}^{-1}$) from the 72% degradation data.

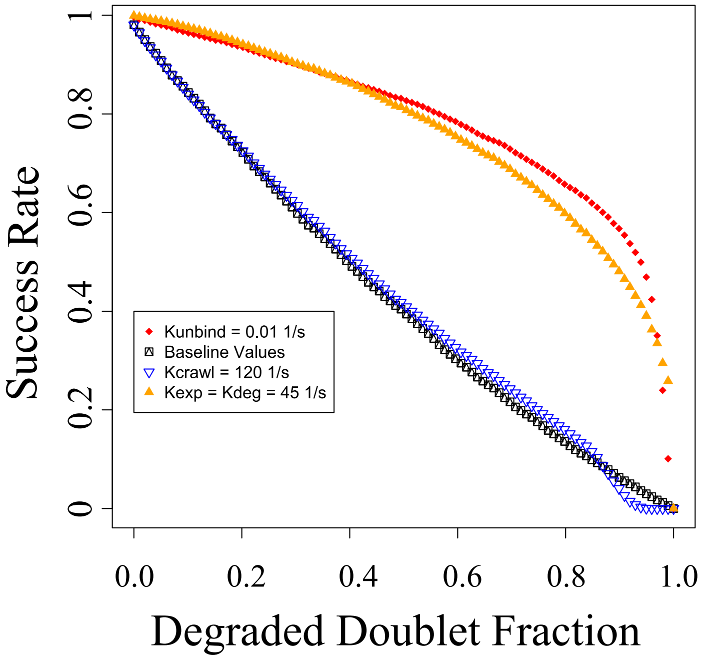

Fig. A Success Rate. Plot of the success rate (fraction of runs out of 10,000 for which cleavage occurred) as a function of degraded doublet fraction for all 4 parameter sets. All parameter values are held fixed at the baseline values listed in Table 1 except for the following: $k_{\mathrm{unbind}} = 0.01\ \mathrm{s}^{-1}$ (red diamond), $k_{\mathrm{krawl}} = 120\ \mathrm{s}^{-1}$ (empty blue triangle), $k_{\mathrm{exp}} = k_{\mathrm{deg}} = 45\ \mathrm{s}^{-1}$ (solid orange triangle).

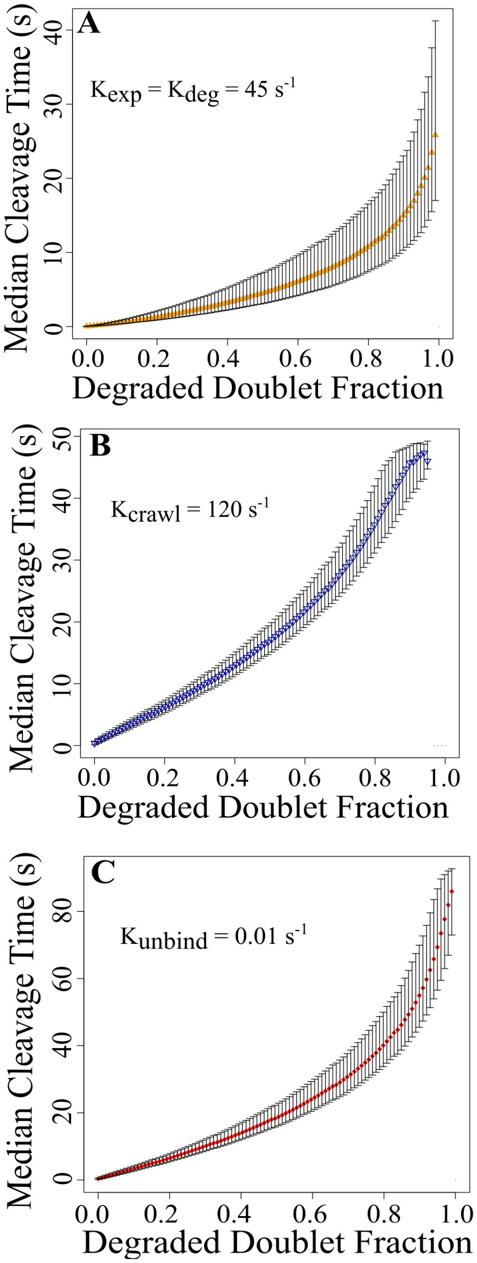

Fig. B Median cleavage time at different degraded doublet fractions. The results are obtained from 10,000 simulations. The top and bottom error bars show the 95th and 5th percentiles, respectively. A: Data for $k_{\mathrm{exp}} = k_{\mathrm{deg}} = 45\ \mathrm{s}^{-1}$. B: Data for $k_{\mathrm{krawl}} = 120\ \mathrm{s}^{-1}$. C: Data for $k_{\mathrm{unbind}} = 0.01\ \mathrm{s}^{-1}$.

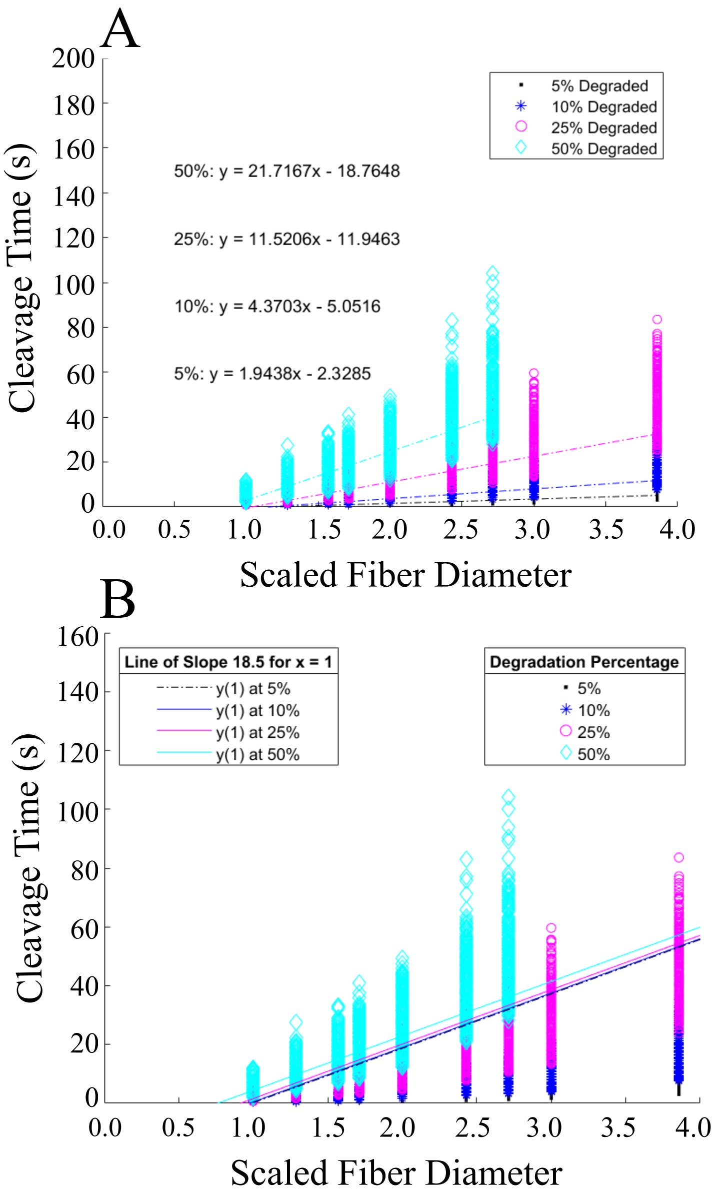

Fig. C Cleavage time as a function of scaled fiber diameter for higher exposure and degradation rate data. Parameter values were set at their baseline rates given in Table 1, except that $k_{\mathrm{exp}} = k_{\mathrm{deg}} = 45\ \mathrm{s}^{-1}$. A: Model data showing cleavage time as a function of the scaled fiber diameter. cleavage was defined as when 0.05 (black, 5%), 0.10 (blue, 10%), 0.25 (pink, 25%), or 0.5 (cyan, 50%) of the doublets in the cross-section had been degraded. Lines of best fit were computed for each of those four different sets of data. B: The same model data as in A, but with lines of slope 18.5 passing through the median cleavage time at scaled fiber diameter 1.0.
